# Supplementary material for: An open-source framework for large-scale, flexible evaluation of biomedical text mining systems
Source: J Biomed Discov Collab. 2008 Jan 29;3:1. doi: 10.1186/1747-5333-3-1 (PMC2276192; doi:10.1186/1747-5333-3-1)
Supplement: Additional file 1 — Evaluation platform source code. The additional file contains the software evaluation framework discussed in this paper. The most current release can be downloaded from SourceForge.net [1]. See the accompanying README file for instructions on installation and use. Also included in the distribution is a collection of UIMA wrappers for some commonly used BioNLP tools and annotated corpora. [file 1747-5333-3-1-S1.zip › bionlp_uima_repository-1.0.1/docs/api/src-html/edu/uchsc/ccp/uima/ae/nlp/sentenceDetector/KeXSentenceDetector_AE.html]

```
001    /*
002     * Created on Aug 15, 2005
003     *
004     * I created this for Andy to test out SIAPI
005     */
006    package edu.uchsc.ccp.uima.ae.nlp.sentenceDetector;
007    
008    
009    import java.util.Iterator;
010    import java.util.List;
011    
012    import org.apache.uima.analysis_engine.ResultSpecification;
013    import org.apache.uima.analysis_engine.annotator.AnnotatorConfigurationException;
014    import org.apache.uima.analysis_engine.annotator.AnnotatorContext;
015    import org.apache.uima.analysis_engine.annotator.AnnotatorContextException;
016    import org.apache.uima.analysis_engine.annotator.AnnotatorInitializationException;
017    import org.apache.uima.analysis_engine.annotator.AnnotatorProcessException;
018    import org.apache.uima.analysis_engine.annotator.JTextAnnotator_ImplBase;
019    import org.apache.uima.jcas.JCas;
020    
021    import edu.uchsc.ccp.uima.annotation.CCPDocumentInformation;
022    import edu.uchsc.ccp.uima.util.UIMA_Util;
023    import edu.uchsc.ccp.util.nlp.GenericTextUtils;
024    import edu.uchsc.ccp.util.nlp.annotation.TextAnnotation;
025    
026    /**
027     * @author williamb
028     *
029     */
030    public class KeXSentenceDetector_AE extends JTextAnnotator_ImplBase {
031            
032            
033            public static final String PARAM_KEXBASEDIRECTORY = "KeXBaseDirectory"; 
034        
035        private final int annotatorID = 3;
036            private final boolean DEBUG = false;
037        
038            private String kexBaseDirectory;
039            
040              public void initialize(AnnotatorContext arg0) throws AnnotatorInitializationException, AnnotatorConfigurationException {
041                 try {
042                  this.kexBaseDirectory = (String)arg0.getConfigParameterValue(PARAM_KEXBASEDIRECTORY);
043                    } catch (AnnotatorContextException e) {
044                            System.err.println("Error reading parameters from SentenceTokenizer descriptor file...");
045                            e.printStackTrace();
046                    }
047                    super.initialize(arg0);
048                }
049            
050            
051            /* (non-Javadoc)
052             * @see com.ibm.uima.analysis_engine.annotator.JTextAnnotator#process(com.ibm.uima.jcas.impl.JCas, com.ibm.uima.analysis_engine.ResultSpecification)
053             */
054            public void process(JCas jcas, ResultSpecification arg1) throws AnnotatorProcessException {
055    
056            /* get document ID */
057            Iterator docInfoIter = jcas.getJFSIndexRepository().getAnnotationIndex(CCPDocumentInformation.type).iterator();
058            String documentID = "-1";
059            if (docInfoIter.hasNext()) {
060                CCPDocumentInformation srcDocInfo = (CCPDocumentInformation) docInfoIter.next();
061                documentID = srcDocInfo.getDocumentID();
062            }
063            
064                    /* get document text */
065                    String docText = jcas.getDocumentText();
066            
067                    /* tokenized into sentences and words using oneline (part of the KeX system) */
068                    List<TextAnnotation> sentences;
069            
070                sentences = GenericTextUtils.tokenizeIntoSentences(kexBaseDirectory,docText, documentID);
071                
072                /* add protein annotations to the CAS */
073                UIMA_Util uimaUtil = new UIMA_Util();
074                uimaUtil.putTextAnnotationsIntoJCas(jcas, sentences);
075            
076                    
077    
078            
079                    
080            }
081            
082            
083      
084    }
```
